# Albumin Binding Function: The Potential Earliest Indicator for Liver Function Damage

**DOI:** 10.1155/2016/5120760

**Published:** 2016-12-22

**Authors:** Penglei Ge, Huayu Yang, Jingfen Lu, Wenjun Liao, Shunda Du, Yingli Xu, Haifeng Xu, Haitao Zhao, Xin Lu, Xinting Sang, Shouxian Zhong, Jiefu Huang, Yilei Mao

**Affiliations:** ^1^Department of Liver Surgery, Peking Union Medical College (PUMC) Hospital, Chinese Academy of Medical Sciences and PUMC, Beijing, China; ^2^State Key Laboratory of Natural and Biomimetic Drugs, School of Pharmaceutical Sciences, Peking University, Beijing, China

## Abstract

*Background*. Currently there is no indicator that can evaluate actual liver lesion for early stages of viral hepatitis, nonalcoholic fatty liver disease (NAFLD), and cirrhosis. Aim of this study was to investigate if albumin binding function could better reflect liver function in these liver diseases.* Methods*. An observational study was performed on 193 patients with early NAFLD, viral hepatitis, and cirrhosis. Cirrhosis patients were separated according to Child-Pugh score into A, B, and C subgroup. Albumin metal ion binding capacity (Ischemia-modified albumin transformed, IMAT) and fatty acid binding capacity (total binding sites, TBS) were detected.* Results*. Both IMAT and TBS were significantly decreased in patients with NAFLD and early hepatitis. In hepatitis group, they declined prior to changes of liver enzymes. IMAT was significantly higher in cirrhosis Child-Pugh class A group than hepatitis patients and decreased in Child-Pugh class B and class C patients. Both IMAT/albumin and TBS/albumin decreased significantly in hepatitis and NAFLD group patients.* Conclusions*. This is the first study to discover changes of albumin metal ion and fatty acid binding capacities prior to conventional biomarkers for liver damage in early stage of liver diseases. They may become potential earliest sensitive indicators for liver function evaluation.

## 1. Introduction

Viral hepatitis in some countries, especially Asian countries, has an incidence rate that is 8% or higher [1]. It is recognized as an important contributive factor for cirrhosis and hepatocellular carcinoma [2, 3]. The currently conventional biochemical markers for liver function, such as bilirubin, albumin, and liver enzymatic assays, often do not exhibit detectable changes at early stage of viral hepatitis [4]. This makes early detection, assessment, and timely treatment of this disease very difficult. In most cases, liver fibrosis or cirrhosis has already occurred when these indicators become abnormal. NAFLD is becoming a common chronic disease in both developed and developing countries with increasing incidence [5–7]. Nonalcoholic steatohepatitis (NASH) may also cause cirrhosis and eventually lead to hepatocyte carcinoma [8, 9]. Conventional liver function tests are not able to assess the liver damages at the early stages of the diseases; effective markers for early liver damages are needed.

Serum albumin, the most abundant circulating proteins in the body, plays a very important role in maintaining the osmotic pressure in blood vessels. In addition, it has binding and transportation functions [10, 11]. However, most of the studies focused on the importance of serum albumin levels instead of its binding functions. Albumin binding functions include its metal ion binding and fatty acid binding. Metal ion binding function reflects the binding capacity of the N-terminal metal ion binding site to metal ions. Fatty acid binding function is the binding capacity for long-chain fatty acids that can be detected with electron paramagnetic resonance (EPR) technique [12, 13].

In recent years, some studies have been published related to the significance of albumin binding functions. Jalan et al. [14] found that albumin fatty acid binding ability and metal ion binding capacity were significantly lower in advanced cirrhosis, which can be used to predict prognosis of acute on chronic liver failures. Oettl et al. [15] reported that in patients with advanced liver disease, the reduction of binding capacity at albumin binding site II that binds to aromatic carboxylic compounds is related to impaired liver function. However, there were no reports about the changes in albumin binding activities in the early stages of viral hepatitis or nonalcoholic fatty liver disease (NAFLD). In the present observational study, we observed for the first time that albumin cobalt binding capacity and albumin fatty acid binding capacity changed prior to any changes observed in other conventional liver function assays; and the extents of the changes were associated with the degrees of liver damage. Therefore, these may become new markers for early hepatic dysfunction.

## 2. Materials and Methods

### 2.1. Patients

Patients were enrolled from January 2014 to February 2015 at this hospital, among whom are NAFLD (*n* = 23), viral hepatitis (*n* = 37), and cirrhosis (*n* = 133) [6, 8, 16] (Table 1). Cirrhosis patients were further divided according to Child-Pugh score [17] into three subgroups, Child-Pugh class A had 81 patients, Child-Pugh class B had 30 patients, and Child-Pugh class C had 22 patients. Another 60 healthy volunteers were also enrolled as control. The study was approved by the ethics committee of Peking Union Medical College Hospital. All subjects were provided with written informed consent in accordance with the Declaration of Helsinki prior to their inclusion in the study.

### 2.2. Serum Sample Collection

Peripheral venous blood was collected after at least 12 hours of fasting. Blood samples were collected into the additive-free vacuum tube and allowed 30–60 minutes for coagulation. The samples were then centrifuged at 3000 revolutions per minute for 10 minutes, and serum was stored at −80°C before analysis.

### 2.3. Routine Serological Tests

Routine tests included total bilirubin, aspartate aminotransferase (AST), alanine aminotransferase (ALT), creatinine, prothrombin time (PT), and international normalized ratio (INR). Child-Pugh score and model for end stage liver disease (MELD) score were also computed [17].

### 2.4. Albumin Binding Activity Assay

#### 2.4.1. Albumin Cobalt Binding Capacity

The procedure was based on prior description by Bar-Or et al. [18]. In short, 100 *μ*L of serum was mixed with 25 *μ*L cobalt chloride (CoCl_2_, 1 mg/mL) (Sigma-Aldrich, USA) in a 96-well plate and incubated at room temperature for 10 minutes. 25 *μ*L dithiothreitol (DTT, 1.5 mg/mL) (Sigma-Aldrich, USA) was added, followed by 2-minute incubation to allow the reaction with free cobalt salt. Finally 150 *μ*L saline was added to terminate the reaction. Absorbance was measured by a spectrophotometer Synergy H1 (BioTek, USA) at 470 nm. The IMA was expressed as absorbance unit, which equaled the absorbance of the testing well minus the absorbance of control well with no DTT added. High absorbance value indicated more free cobalt salt reacting with DTT; therefore fewer cobalt salt was bound to albumin.

#### 2.4.2. Albumin Fatty Acid Binding Capacity

Serum (100 *μ*L) was transferred into 0.5 mL of an Eppendorf tube and 3.0 mmol/L of spin label, 16-doxyl stearic acid (Sigma-Aldrich, USA), was added. The mixture was gently stirred with a 2 mm diameter glass rod for 3 minutes at 4°C. The Eppendorf tube was then sealed and placed in a water bath at 37°C and incubated for 20 minutes. 10 *μ*L of the mixture was then transferred with a quartz capillary onto an electron paramagnetic resonance instrument (Bruker EMX A200, Bruker BioSpin GmbH, German), operated with center field of 3427.4 G, sweep width of 100 G, microwave frequency of 9.6 GHz, microwave power of 6.5 MW, and modulation frequency of 100 KHz with modulation amplitude 2.5 G.

**Figure 1:**
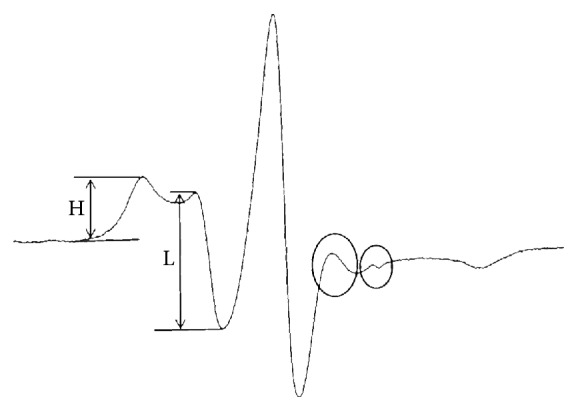
EPR spectrum of the 16-DS bound to albumin and with some unbounded 16-DS in serum. Spin labels bounded at albumin binding sites could be released free to the serum when albumin structure was impaired. The large circle part represents the low affinity binding sites to fatty acid in albumin at *I* = +1. The small circle part represents the free label in serum at *I* = +1. The area of the two circles should be deducted when calculating TBS. H and L, respectively, represent the number of 16-DS bounded to high and low affinity fatty acid binding sites. H/L reflects the fatty acid binding affinity of albumin.

The obtained data was analyzed with the Origin 8 image processing software (OriginLab, USA) to construct a spectral diagram (Figure 1). This was a spectral diagram constructed from the data generated on the electron paramagnetic resonance spectrometer of the spin label and albumin complex. Total area under the spectrum represented the sum of the serum albumin bound spin label and the free label [12], which was calculated by integration.

The spin label, 16-doxyl stearic acid, had very high binding constant to albumin [12]. Thus, normally there were very few free labels present in the serum. However, when albumin structure was impaired, spin labels that were albumin bound at their binding sites had been released free and, therefore, could be detected and recorded on the spectrum. The effect of free spin label in serum on the total fatty acid binding sites of albumin was determined through simulation experiments. Then, TBS, the sum of the binding sites on albumin molecules that were capable of fatty acid binding, was calculated by deducting the free spin labels in serum from the total amount of labels. This also reflected the total amount of functional albumin.

Meanwhile, the ratio between the number of fatty acid binding sites with high-affinity and that with low affinity could also be measured and calculated from the spectrum (H/L). H/L reflected not only the binding strength of albumin to fatty acid, but also the changes in its conformation.

### 2.5. Statistical Analysis

Continuous variables were expressed as mean ± standard deviation, or median and interquartile. Statistical analysis was performed with SPSS 13.0 statistical software (SPSS Inc., USA). Nonparametric Kruskal-Wallis test was used for paired comparisons between data sets with different sample sizes. Two-way test was used for all analysis. *P* < 0.01 was considered statistically significant.

Correlation analysis between groups of data was done with Pearson product-moment correlation coefficient if both variables followed a normal distribution, or with Spearman's rank-order correlation coefficient if one of them did not follow a normal distribution, or between the ranked variables.

## 3. Results

All hepatitis patients were infected with either hepatitis B or C virus, where 78.4% (29/37) of them were hepatitis B patients. Hepatitis B or hepatitis C virus infection was the cause for all cirrhosis, in which hepatitis B accounted for 91.0% (121/133). All patients infected with hepatitis B virus in hepatitis group and cirrhosis group received antiviral therapy. The ratios of patients receiving continuous interferon and nucleos(t)ide analogue therapies for at least six months to that receiving irregular antiviral therapy less than six months in hepatitis, Child-Pugh classes A, B, and C groups were, respectively, 20 : 9, 54 : 21, 19 : 9, and 10 : 8.

No differences were observed in albumin levels among the healthy control and NAFLD and hepatitis patients (*P* = 0.36). However, the albumin levels of cirrhotic patients were significantly lower than those of the noncirrhotic patients (*P* < 0.001). Furthermore, the albumin levels continued to decrease as the liver lost more function. At the same time, clinical Child-Pugh scores and MELD scores of Child-Pugh class B and class C group patients are higher than those of Child-Pugh class A group patients (*P* < 0.01). PT gradually increased, from patients with viral hepatitis to cirrhosis, indicating the decline of liver synthetic function (Table 2).

### 3.1. Albumin Cobalt Binding Capacity

The direct reading of IMA assay reflected the serum albumin that had lost metal ion binding ability. To simplify the presentation, we converted it into IMAT (IMA transformed), which is 1-IMA. Thus, the higher value of IMAT indicated that the tested albumin had higher metal ion binding capacity. There were no differences in serum albumin levels in noncirrhotic patients (NAFLD or viral hepatitis) and control healthy volunteers. However, IMAT values in NAFLD as well as hepatitis patients were lower than the healthy people (*P* < 0.001). There was no difference in IMAT values between the NAFLD and hepatitis patients (*P* = 0.48). In cirrhotic patients, however, higher IMAT values were found in patients in Child-Pugh class A comparing to the NAFLD or hepatitis patients. As the condition of liver becames further deteriorated, the levels of serum albumin decreased as well as the IMAT values (Table 3).

Since the serum albumin levels changed during disease progression, it is perceivable that it may influence the IMAT value tested. To avoid this, we normalized the IMAT against albumin concentration and expressed it as IMAT/albumin. After normalization, similar results were found in which IMAT/albumin levels in NAFLD or hepatitis patients were significantly lower than the healthy volunteers (*P* < 0.001); there were no differences between these two groups of patients (*P* = 0.70). Due to the significant decreases in serum albumin levels seen in cirrhosis patients, the IMAT/albumin values were greatly increased in patients of Child-Pugh classes A and B comparing to that of the hepatitis patients. Their IMAT/albumin values were not different than the healthy control (*P* = 0.07). However, patients of Child-Pugh class C had significant lower IMAT/albumin values comparing to that of the healthy control (*P* < 0.001) (Table 3).

### 3.2. Albumin Fatty Acid Binding Capacity

The ability of albumin in binding fatty acids was assessed by EPR and expressed as TBS. Even though none of the NAFLD or hepatitis patients had yet developed cirrhosis and there were no significant changes in their serum albumin levels in comparison to the healthy people, their TBS values had already shown various degrees of decline. Among them, hepatitis patients had a more significant decline over the healthy control (*P* < 0.001) than the NAFLD patients (*P* = 0.11). The observations in cirrhosis patients were more complicated. Although the serum albumin levels in patients of Child-Pugh class A were significantly lower than that of the hepatitis patients (*P* < 0.001), there were no differences in their TBS values (*P* = 0.31). Increased TBS values were seen in the patients of Child-Pugh class B compared to that in the patients of Child-Pugh class A (*P* < 0.001) (Table 3).

Similarly, we normalized TBS value against the serum albumin concentration. Both NAFLD and hepatitis patients showed significantly decreased TBS/albumin values compared to that of the healthy volunteers (*P* < 0.001). Liver became more dysfunctional during cirrhosis; instead, TBS/albumin values of these patients increased. We observed a minimum increase in Child-Pugh class A patients and a significant increase in Child-Pugh class B and class C patients (*P* < 0.01). There were no statistical differences between class B and class C patients (*P* = 0.89) (Table 3).

### 3.3. Fatty Acid Binding Affinity of Albumin

The binding affinity of each fatty acid binding sites is also part of the binding capacity of albumin, which can be demonstrated as the ratio between high and low affinity binding sites (H/L). To certain extend, this ratio can also imply the changes in albumin protein structure. The results shown in Table 3 indicated that all patients had various declines in H/L compared to that of healthy volunteers. Hepatitis patients as well as cirrhosis patients of Child-Pugh class B and class C all showed significant decrease in H/L compared to healthy control (*P* < 0.001). Patients of Child-Pugh class A did not have significantly different H/L as hepatitis patients (*P* = 0.16). However, as cirrhosis progressed, H/L gradually declined.

### 3.4. Correlation Analysis

Correlation analysis showed that a moderate positive correlation could be found between albumin levels and IMAT, *r* = 0.41 (*P* < 0.001). There was a weak correlation between the H/L and IMAT, *r* = 0.34 (*P* < 0.001), but no correlation between TBS and albumin levels (*P* = 0.13).

## 4. Discussion

Viral hepatitis is one of the main causes for liver cirrhosis and cancer. In addition, the incidents of NAFLD are on the rise in recent years, while the patients suffering from liver cirrhosis and even cancers due to NASH are also increased [8, 19]. The accurate evaluation of liver function and the extent of the damage in the early disease course can help the physicians to devise a reasonable treatment plan promptly to better control the disease development. Thus, it is very important to assess liver damage in the beginning stages of the disease even before the changes in liver enzyme activities become detectable. However, there are limitations in the conventional detection indexes used to assess early liver lesions [4].

The level of serum albumin is one of the indicators for liver synthetic function [17, 20]. However, albumin level itself does not reflect its binding activities, which may be the more sensitive indication for functionality. This study revealed for the first time that there were changes in the albumin binding activities in NAFLD, viral hepatitis, and liver cirrhosis patients; and our findings suggested that albumin binding activity may be an early marker for liver function during disease development comparing to other liver parameters.

Higher value of IMAT indicates greater metal ion binding capacity of albumin. Our results showed that, in NAFLD and viral hepatitis patients, even before their albumin levels, total bilirubin, and prothrombin time became abnormal, their IMAT value decreased significantly comparing to the healthy volunteers (*P* < 0.001). This suggested that IMAT was an earlier and more sensitive indicator for liver dysfunction than the conventional liver biomarkers. In the liver cirrhosis patients, however, we had a more interesting observation. The IMAT values increased in Child-Pugh class A patients comparing to the viral hepatitis patients (*P* < 0.01) but decreased in class B patients and further more in class C patients. It is possible that the Child-Pugh class A patients were in the stage that their livers were trying to compensate the lost function; thus, although the albumin levels decreased in these patients, their ion binding activities increased. This suggested that, during this stage, the albumin levels did not correlate to their binding capacities. However, as the liver function continued to deteriorate, the compensatory increase in binding capacity became lost and the IMAT value started to decrease from patients in Child-Pugh class B to class C.

We used the normalized IMAT/albumin, representing the ion binding capacity in unit albumin molecule, to minimize the influence of fluctuations in serum albumin levels. Results showed that albumin binding capacity per unit albumin in patients of NAFLD and hepatitis decreased significantly compared to normal control (*P* < 0.001). During the liver functional compensation period in patients of cirrhosis, IMAT/albumin also has a short compensation period and then declined progressively with the deterioration of liver function.

The reason that the binding capacity of albumin changes in the early stage of liver damage is still unclear. It is possible that during hepatocytes steatosis, inflammation response, or viral infection, the three-dimensional structure of the albumin molecule is altered, leading to the structural changes on its metal ion binding site and the reduction of its ion binding capacity. In the early cirrhosis, there is limited compensation for albumin ion binding capacity.

The fatty acid binding ability of albumin is directly associated with the correct structure at its fatty acid binding sites. Therefore, the normal fatty acid binding capacity can only be maintained in structurally normal albumin. Studies have found that there are seven fatty acid binding sites on albumin, in which three are high-affinity and four are low affinity binding sites [12]. The spin labeling electron paramagnetic resonance spectroscopy enabled us to detect not only the number of total binding sites but also the binding affinity of albumin to fatty acid. We found that, in the early stages of NAFLD and hepatitis, there were already various extents of damage to albumin's fatty acid binding capacity, in which hepatitis patients suffered more severe damages. Normalized TBS/albumin represented the number of binding sites per unit of albumin. Our results showed that TBS/albumin value decreased significantly in NAFLD and hepatitis patients over the healthy volunteers (*P* < 0.01). Compared to conventional biochemical markers, TBS may be a more effective early indicator for liver damage. In the later stage of cirrhosis, when albumin levels had shown significant decline, their fatty acid binding capacity was still in a compensation stage. TBS/albumin values showed a compensatory increase in patients with further declined liver function. TBS/albumin values were both higher in patients in Child-Pugh class B and class C compared to that of the patients in class A (*P* < 0.001). As cirrhosis became more severe, the fatty acid binding capacity of albumin gradually declined. The patients in Child-Pugh class C lost the compensatory increase of fatty acid binding capacity of their albumins.

We try to explain the possible reasons for this outcome in light of three-dimensional structural changes in albumin. The ratio between high-affinity and low affinity binding sites (H/L) also indicates the albumin conformational changes in addition to albumin binding ability. Our results showed that albumin conformation was damaged in NAFLD and hepatitis patients, while the damage was more severe in hepatitis patients. Accordingly, the TBS values of hepatitis patients were lower than that of NAFLD patients. As disease progressed to cirrhosis, compensatory increases of TBS/albumin were seen to overcome the adverse effect of decreased albumin levels. Once the structural damage of albumin became more severe, the values of TBS/albumin stopped to increase and TBS started to decline.

Both metal ion binding and fatty acid binding capacities of albumin reflected liver damage earlier than the changes in albumin levels. There is no correlation between the changes of IMAT and TBS, suggesting that there were differential effects on various ligand-binding sites of albumin in different diseases that impaired liver functions. The exact mechanism of compensatory binding function increase seen in albumin remains unclear. In addition, it is complex for the changing of albumin binding function in patients with cirrhosis, so further studies are needed to probe the internal mechanism.

## 5. Conclusions

Currently, there are no effective markers for detecting liver damages in the early stages of viral hepatitis and NAFLD. In conclusion, this study is the first to discover that the metal ion binding and fatty acid binding capacities of albumin had undergone significant change at this early stage. They may potentially become earliest sensitive indicators to suggest liver dysfunction, which could have positive impacts on evaluation and treatment of early stage liver diseases.

One limitation of the study is the small sample size. Furthermore, because antiviral treatment can give a significant effect on liver function in patients with hepatitis and reduce the cirrhosis decompensation, treatment protocols may affect the results of albumin binding function.

## Figures and Tables

**Table 1:** Inclusion and exclusion criteria for NAFLD, hepatitis, and cirrhosis patients.

NAFLD	Inclusion criteria	No alcohol history or consuming of alcohol < 210 g/week for male and < 140 g/week for female
		Ultrasound, CT, MRI, or liver biopsy prompted fatty liver

NAFLD	Exclusion criteria	Viral hepatitis, drug-induced hepatitis, alcoholic fatty liver, autoimmune liver disease, liver degeneration
		Diagnosis of cirrhosis by ultrasound, CT, MRI, or liver biopsy pathology; neoplasm in liver or other organs
		With myocardial ischemia, hepatorenal syndrome, diabetes, infections, intestinal ischemia, cerebrovascular accident
		Albumin infusion within a month

Hepatitis	Inclusion criteria	Viral hepatitis over six months
		HBsAg and/or HBeAg positive
		Ultrasound, CT, MRI, or liver biopsy prompted no liver cirrhosis; AST, ALT < 40 U/L

Hepatitis	Exclusion criteria	Neoplasm in liver or other organs
		With myocardial ischemia, hepatorenal syndrome, diabetes, infections, intestinal ischemia, cerebrovascular accident
		Albumin infusion within a month

Cirrhosis	Inclusion criteria	Diagnosis of cirrhosis by ultrasound, CT, MRI, liver biopsy pathology, or operation

Cirrhosis	Exclusion criteria	Neoplasm in liver or other organs
		With myocardial ischemia, hepatorenal syndrome, diabetes, infections, intestinal ischemia, cerebrovascular accident
		Alcoholic, nonalcoholic, drug-induced, primary biliary cirrhosis, and unexplained hepatic cirrhosis albumin infusion within a month

**Table 2:** Baseline characteristics of the participants in different subjects groups.

Parameters	Control (*n* = 60)	NAFLD (*n* = 23)	Hepatitis (*n* = 37)	Cirrhosis		
				Child-Pugh class A	Child-Pugh class B	Child-Pugh class C
				(*n* = 81)	(*n* = 30)	(*n* = 22)
Age (yr)	47.4 ± 9.7	43.5 ± 11.4	47.0 ± 10.6	49.0 ± 12.8	50.7 ± 11.7	56.1 ± 9.2
Sex (M/F)	32/28	17/6	22/15	75/6	21/9	16/6
Viral hepatitis (hbv/hcv)	No	No	29/8	75/6	28/2	18/4
Albumin (g/L)	46.7 ± 3.6	48.0 ± 2.6	47.0 ± 3.1	41.3 ± 5.2^*∗*#*⊿*^	33.1 ± 4.1^*∗*#*⊿*†^	30.0 ± 5.2^*∗*#*⊿*†^
Bilirubin (*μ*mol/L)						
Median	11.7	12.5	12.4	15.6^*∗*^	21.1^*∗*#*⊿*^	114.1^*∗*#*⊿*†§^
Interquartile range	9.0–15.2	8.0–17.3	10.9–16.3	11.6–21.7	12.3–29.7	52.0–308.6
AST (U/L)						
Median	17.5	40.0^*∗*^	24.0^#^	25.9^*∗*#^	37.5^*∗⊿*^	47.3^*∗⊿*†^
Interquartile range	14.0–20.8	36.0–57.0	20.0–26.0	21.0–40.0	29.0–62.3	38.0–120.0
ALT (U/L)						
Median	17.5	66.0^*∗*^	20.0^#^	26.0^*∗*#*⊿*^	34.5^*∗⊿*^	24.7^#^
Interquartile range	12.5–24.0	58.0–95.0	15.0–25.0	17.5–39.5	26.8–43.0	18.0–75.2
Creatinine (*μ*mol/L)	68.5 ± 12.1	70.1 ± 17.9	72.0 ± 12.3	74.1 ± 14.1	73.9 ± 17.4	84.1 ± 13.8^*∗⊿*^
PT (s)	11.5 ± 0.6	11.5 ± 0.7	11.6 ± 0.6	12.3 ± 1.3^*∗*^	13.5 ± 1.4^*∗*#*⊿*†^	19.4 ± 5.5^*∗*#*⊿*†^
INR	1.0 ± 0.1	1.0 ± 0.1	1.0 ± 0.1	1.1 ± 0.2^*∗*#*⊿*^	1.2 ± 0.1^*∗*#*⊿*†^	1.7 ± 0.5^*∗*#*⊿*†^
Child-Pugh score	n.a.	n.a.	n.a.	5.2 ± 0.4	7.5 ± 0.7^†^	11.0 ± 1.2^†^
MELD score	n.a.	n.a.	n.a.	7.9 ± 1.9	10.5 ± 3.1^†^	21.3 ± 12.7^†^

Continuous variables are expressed as means ± standard deviation, or median and interquartile. M: male; F: female; hbv: hepatitis B virus; hcv: hepatitis C virus; n.a.: not available; MELD: model for end stage liver disease.

^*∗*^ *P* < 0.01 compared with control group; ^#^ *P* < 0.01 compared with NAFLD group.

^*⊿*^ *P* < 0.01 compared with hepatitis group; ^†^ *P* < 0.01 compared with Child-Pugh class A group.

^§^ *P* < 0.01 compared with Child-Pugh class B group.

**Table 3:** Functional albumin parameters in different subjects groups.

Parameters	Control (*n* = 60)	NAFLD (*n* = 23)	Hepatitis (*n* = 37)	Cirrhosis		
				Child-Pugh class A	Child-Pugh class B	Child-Pugh class C
				(*n* = 81)	(*n* = 30)	(*n* = 22)
IMAT	0.59 ± 0.06	0.42 ± 0.16^*∗*^	0.41 ± 0.12^*∗*^	0.51 ± 0.13^*∗⊿*^	0.38 ± 0.12^*∗*†^	0.22 ± 0.06^*∗*#*⊿*^
IMAT/albumin (10^−2^)	1.27 ± 0.15	0.88 ± 0.32^*∗*^	0.87 ± 0.24^*∗*^	1.25 ± 0.32^#*⊿*^	1.15 ± 0.35^*⊿*^	0.77 ± 0.22^*∗*†§^
TBS (10^8^)	6.03 ± 0.36	5.69 ± 0.31	5.42 ± 0.22^*∗*^	5.34 ± 0.32^*∗*#^	5.97 ± 0.58^*⊿*†^	5.48 ± 0.46^*∗*§^
TBS/albumin (10^7^)	1.30 ± 0.14	1.19 ± 0.09^*∗*^	1.18 ± 0.12^*∗*^	1.29 ± 0.15	1.86 ± 0.29^*∗*#*⊿*†^	1.88 ± 0.32^*∗*#*⊿*†^
H/L	0.76 ± 0.08	0.73 ± 0.03	0.69 ± 0.04^*∗*#^	0.72 ± 0.02	0.68 ± 0.04^*∗*#^	0.59 ± 0.10^*∗*#†^

Continuous variables are expressed as means ± standard deviation.

^*∗*^ *P* < 0.01 compared with control group; ^#^ *P* < 0.01 compared with NAFLD group.

^*⊿*^ *P* < 0.01 compared with hepatitis group; ^†^ *P* < 0.01 compared with Child-Pugh class A group.

^§^ *P* < 0.01 compared with Child-Pugh class B group.

## Abbreviations

NAFLD: Nonalcoholic fatty liver disease
IMA: Ischemia-modified albumin
IMAT: Ischemia-modified albumin transformed
TBS: Total binding sites
NASH: Nonalcoholic steatohepatitis
EPR: Electron paramagnetic resonance
AST: Aspartate aminotransferase
ALT: Alanine aminotransferase
PT: Prothrombin time
INR: International normalized ratio
MELD: Model for end stage liver disease
DTT: Dithiothreitol.
